# Supplementary figures and images for: A dendritic guidance receptor functions in both ligand dependent and independent modes
Source: bioRxiv. 2025 Feb 12:2025.02.11.637350. Preprint. [Version 1] doi: 10.1101/2025.02.11.637350 (PMC11844549; doi:10.1101/2025.02.11.637350)

## Supplementary Figure 1

A

10 mM  
Auxin

DMA-1::GFP::AID

-

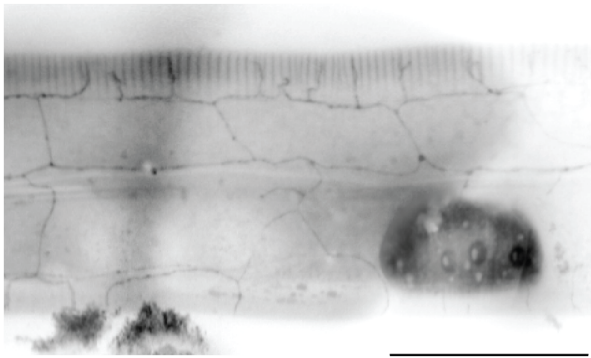

+

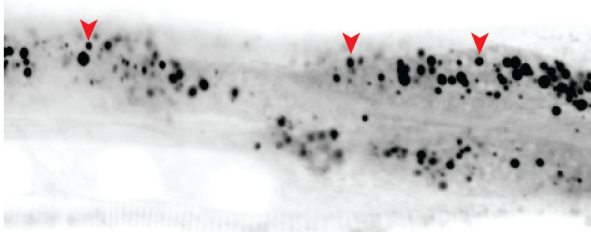

B

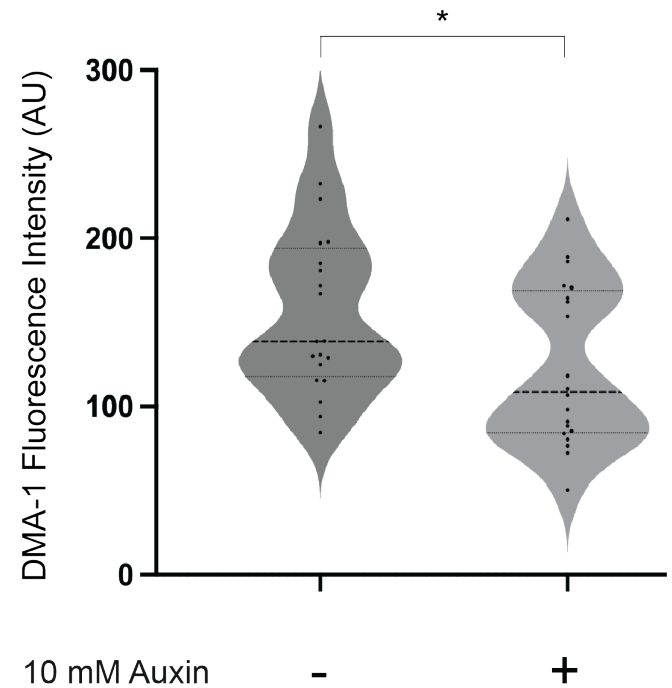

Supplement: 1 — Supplementary Figure 1. Temporal degradation of degron tagged DMA-1. (A) Lateral fluorescence sum intensity z-projections of endogenously labelled DMA-1::GFP::AID treated without auxin (top) or with 10 mM Auxin (bottom). Scale bar, 50 μm. Red arrowheads indicate examples of gut granules which exhibit autofluorescence and are not present in the PVD neuron (B) Quantifications of DMA-1::GFP::AID fluorescence intensity in the PVD cell body. Medians are represented in thick dashed lines and quartiles are represented in thin dashed lines. P value was calculated using a two-tailed unpaired Student’s t-test. n=20 for all conditions. *p≤0.05. [file NIHPP2025.02.11.637350V1-supplement-1.pdf]
